# Diagnosing multiple sclerosis: implementation and unintended effects of the 2024 McDonald criteria

**DOI:** 10.1093/brain/awag293

**Published:** 2026-08-28

**Authors:** Michael A Foster, Charles Wade, Helen L Ford, Klaus Schmierer

**Affiliations:** Queen Square MS Centre, Department of Neuroinflammation, UCL Queen Square Institute of Neurology, Faculty of Brain Sciences, University College London, London WC1B 5EH, UK; Department of Neurology, Queen’s Hospital, Barking, Havering and Redbridge University Hospitals NHS Trust, Romford RM7 0AG, UK; Queen Square MS Centre, Department of Neuroinflammation, UCL Queen Square Institute of Neurology, Faculty of Brain Sciences, University College London, London WC1B 5EH, UK; Department of Neurology, National Hospital for Neurology and Neurosurgery, University College London Hospitals NHS Foundation Trust, London WC1N 3BG, UK; School of Medicine, Faculty of Health and Life Sciences, University of Leeds, Leeds LS2 9JT, UK; Centre for Neurosciences, Leeds General Infirmary, Leeds Teaching Hospitals NHS Trust, Leeds LS1 3EX, UK; Clinical Board Medicine (Neuroscience), The Royal London Hospital, Barts Health NHS Trust, London E1 1FR, UK; Centre for Neuroscience, Surgery & Trauma, The Blizard Institute, Faculty of Medicine and Dentistry, Queen Mary University of London, London E1 2AT, UK

**Keywords:** multiple sclerosis, diagnostic criteria

## Abstract

Diagnostic criteria for multiple sclerosis have evolved substantially since the Schumacher criteria were published in 1965. The most recent iteration, the 2024 McDonald diagnostic criteria, was recently released. Each revision has broadened the range of evidence that can support diagnosis while attempting to preserve specificity. The central question raised by the 2024 revision is therefore not simply whether the criteria are valid in principle, but whether they can be applied safely outside expert centres, where imaging quality, laboratory infrastructure and subspecialist interpretation vary substantially.

In this review, we evaluate the 2024 criteria not only as a consensus document but as an intervention in clinical behaviour, with particular attention to misdiagnosis risk, operational feasibility and equity of implementation. In particular, we consider the role of the optic nerve and of the central vein sign and paramagnetic rim lesions in diagnosis, and how the criteria bring more diverse populations and presentations into consideration. We also review how the greater options available to achieve a diagnosis may introduce heterogeneity into diagnostic approaches, and what safeguards can be taken to minimize the risk of inappropriate application of the criteria and subsequent misdiagnosis (and inappropriate treatment). The review finishes by proposing how the changes introduced in this iteration might be refined, with more robust definitions on acquiring and interpreting the novel biomarkers.

## Teaser

Foster *et al*. review the evolution of multiple sclerosis diagnostic criteria and the changes introduced in the 2024 McDonald criteria. They examine how new diagnostic options could increase variation and misdiagnosis risk across healthcare settings, and outline safeguards to support accurate and consistent diagnosis.

**See Evangelou and Gilmartin (https://doi.org/10.1093/brain/awag346) for a scientific commentary on this article.**

## Introduction

Revised diagnostic criteria for multiple sclerosis have recently been published.^1^ Several recent reviews have summarized the content of the 2024 criteria and their practical implementation.^2^ In this structured analytical review, we pay greater attention to how these changes may alter diagnostic behaviour, operational thresholds, and the balance between early diagnosis and misdiagnosis in routine clinical practice.

## The evolution of the multiple sclerosis diagnostic criteria

The first widely adopted diagnostic criteria for multiple sclerosis were the Schumacher criteria, published in 1965.^3^ The criteria set out the requirement that there should be clinical evidence of dissemination in both space and time, that there was evidence of white-matter involvement, and that there should be no better explanation for the patient’s symptoms and signs (a stipulation that has been retained in all subsequent iterations of the diagnostic criteria). The criteria also introduced the concept of clinically definite multiple sclerosis. However, they were developed when the only paraclinical biomarker available to clinicians was evoked potentials (visual or sensory).

Subsequent iterations of diagnostic criteria were able to integrate further biomarkers as they became available. The Poser criteria,^4^ published in 1983, facilitated use of oligoclonal band (OCB) testing in CSF to support a multiple sclerosis diagnosis. The first version of the McDonald criteria^5^ (released in 2001) incorporated MRI findings in the determination of dissemination in space (DIS) and dissemination in time (DIT), while still allowing the presence of CSF-specific OCB to support a diagnosis. It defined the CNS regions that could be assessed for DIS on MRI—lesions needed to be juxtacortical, periventricular, infratentorial or in the spinal cord—and it also included criteria for the diagnosis of primary progressive multiple sclerosis for the first time. The 2005 iteration^6^ clarified how DIT might be achieved on repeat MRI, formalized the inclusion of spinal cord lesions when determining DIS and permitted a diagnosis of primary progressive multiple sclerosis without CSF analysis. The third version,^7^ agreed in 2010, simplified how DIS could be fulfilled (though the symptomatic lesion could not be counted if it were a brainstem or spinal cord presentation). They also allowed the use of a single MRI study demonstrating both gadolinium-enhancing and non-enhancing lesions to fulfil DIT, such that both DIS and DIT criteria could be met with a single set of imaging, though OCB testing was removed from this iteration.

The last significant update before the new 2024 criteria being discussed here was agreed in 2017 and published in 2018.^8^ The use of CSF OCB was reintroduced to reduce the risk of misdiagnosis, such that their presence in CSF but not in serum was an alternative to DIT if DIS was met in a patient presenting with their first demyelinating event. Additionally, when determining DIS, symptomatic lesions now contributed to lesion count, and cortical brain lesions could be included as well as juxtacortical lesions. However, due to a perceived paucity of evidence supporting its inclusion, the optic nerve was not added to the regions permitted for determining DIS, despite it being a well-known site of demyelination and optic neuritis being a common first presentation of multiple sclerosis. Furthermore, the radiologically isolated syndrome, where imaging is typical for multiple sclerosis but without any clear clinical manifestations, was not incorporated into the criteria, and no clear guidelines were given for paediatric-onset or late-onset multiple sclerosis. Though the authors noted the emergence of techniques to distinguish multiple sclerosis lesions from T2 hyperintensities in other conditions, these were similarly not incorporated. These areas were highlighted as important for future research if they were to be considered for future criteria iterations. A summary of the evolution of the diagnostic criteria is in Table 1.

**Table 1:** Evolution of multiple sclerosis diagnostic criteria

Version (reference)	Key features and changes
Schumacher 1965 (Schumacher *et al*.^3^)	First to require DIS and DIT No better explanation for presentation
Poser 1983 (Poser *et al*.^4^)	Added OCB testing
McDonald 2001 (McDonald *et al*.^5^)	Added MRI to diagnostic pathway Defined CNS regions for DIS Added PPMS to criteria
McDonald 2005 (Polman *et al*.^6^)	Clarified DIT with repeat MRI PPMS diagnosis without CSF
McDonald 2010 (Polman *et al*.^7^)	Simplified lesion counts for DIS Restricted use of symptomatic lesions for DIS Single MRI study with gadolinium could fulfil DIT OCB testing removed
McDonald 2017 (Thompson *et al*.^8^)	OCB testing reintroduced (as alternative to DIT) All symptomatic lesions contribute to DIS

DIS = dissemination in space; DIT = dissemination in time; OCB = oligoclonal bands; PPMS = primary progressive multiple sclerosis.

These iterations of the diagnostic criteria have had significant clinical impact. Each successive version is associated with reduced time from first demyelinating event to diagnosis of multiple sclerosis: in one analysis,^9^ median time to multiple sclerosis diagnosis was 20 months from first demyelinating event under the Poser criteria and reduced to 4.6 months with the McDonald 2017 criteria. Similarly, time from first demyelinating event to starting treatment fell from a median of 35.8 months to a median of 6.6 months.

In addition to facilitating earlier accurate diagnosis of multiple sclerosis, diagnostic criteria also consider how to avoid misdiagnosis. Studies of misdiagnosis report rates of 4.4%–30% of people with an apparent established diagnosis of multiple sclerosis.^10-16^ The studied populations vary, in particular by whether the diagnosis of multiple sclerosis had been given by a physician with specialist interest in multiple sclerosis and neuroinflammation, or alternatively by a generalist seeking a subspecialist opinion. Higher rates of misdiagnosis tended to be reported by analyses of referrals to a tertiary centre, rather than reappraisal of diagnoses given by a tertiary centre. There has, however, not been a comprehensive assessment of misdiagnosis rates and how they have changed with updates to the diagnostic criteria.

## Changes in the 2024 revisions

The International Advisory Committee on Clinical Trials in Multiple Sclerosis met through 2022 and 2023, and following a consensus conference in late 2023, presented their new proposed criteria at the 2024 European Committee for Treatment and Research in Multiple Sclerosis conference in Copenhagen (Denmark). The final version of the agreed diagnostic criteria was published in 2025,^1^ and incorporated changes to the determination of DIS, the role of DIT, and the inclusion of new paraclinical biomarkers to facilitate earlier and more accessible diagnosis of multiple sclerosis.

### The optic nerve and dissemination in space

Since the 2017 diagnostic criteria were published, many groups have explored what effect the addition of the optic nerve to DIS criteria would have to diagnostic performance, and the techniques that might be used to determine optic nerve involvement. The optic nerve is a highly relevant area when completing DIS, considering the large proportion of people living with multiple sclerosis for whom an optic neuritis is their first demyelinating event,^17,18^ but for whom the region could not previously be used to facilitate diagnosis of multiple sclerosis. In an assessment in which optic nerve involvement was identified clinically or with visual evoked potentials (VEPs),^19^ the addition of the optic nerve to DIS criteria in patients presenting with an optic neuritis increased the sensitivity (95% versus 83%) and accuracy (81% versus 78%), at the expense of specificity (57% versus 68%); in a larger cohort with VEPs performed in all participants,^20^ optic nerve involvement provided modest improvements in diagnostic sensitivity (82% versus 79%) and accuracy (78% versus 76%) without an impact on specificity (52%). A similar analysis when considering optical coherence tomography (OCT) to define optic nerve involvement^21^ also showed an improvement in sensitivity (84% versus 78%) and accuracy (81% versus 66%) without a loss of specificity (52%). In a cohort in which every participant had optic nerve MRI,^22^ the addition of the optic nerve to DIS criteria increased sensitivity (98% versus 87%) with a loss of specificity (33% versus 73%) and also demonstrated the modified criteria’s ability to facilitate an earlier diagnosis. In a multi-centre study where all participants had at least two modalities of optic nerve assessment,^23^ the inclusion of the optic nerve in DIS criteria showed an increase in sensitivity (93% versus 88%) with slightly lower specificity (80% versus 82%) and preserved accuracy (87% versus 87%).

Consequently, the optic nerve has now been added as a fifth anatomical location for the determination of DIS, which may be determined by VEPs, OCT or optic nerve MRI. VEP demonstration of optic nerve involvement requires both a reduction in peak latency and a difference in interocular latency. On OCT, an inter-eye difference in either the peripapillary retinal nerve fibre layer or composite macular ganglion cell inner plexiform layer thickness needs to be demonstrated. Optic nerve MRI with fat saturation is more sensitive to optic nerve lesions than conventional brain MRI. The authors were careful to note that there should be no better explanation for optic nerve pathology, with particular attention paid to optic perineuritis, chiasmal involvement or longitudinally extensive lesions.^1^

### Dissemination in space, but not in time

Though DIT is felt to be a key requirement for diagnosis of multiple sclerosis, it is not unique to the condition. Many other chronic neurological conditions also demonstrate DIT, such as neuromyelitis optica spectrum disorder and neurosarcoidosis.^24^ Indeed, CSF-restricted OCB can already act as a substitute for DIT.^8^

In an analysis where additional CNS regions were considered for their contribution to DIS, the presence of lesions in increasing numbers of regions increased diagnostic specificity,^22^ with a further study considering the addition of the optic nerve demonstrating specificity of 100% when four out of the five CNS regions contained evidence of demyelination.^25^ As such, the new criteria no longer require demonstration of DIT, if there is evidence of DIS in four out of five typical CNS regions.^1^

### Iron-sensitive imaging

As the authors noted in the 2017 iteration of the diagnostic criteria,^8^ some MRI techniques were becoming available that could differentiate T2 lesions between those caused by demyelination and those by other pathologies. These techniques leveraged iron-sensitive MRI sequences to identify features of demyelinating pathology in white-matter lesions.

#### The central vein sign

The first of these is the central vein sign (CVS). Multiple sclerosis pathology has long been understood to be perivenous,^26^ though imaging techniques have previously struggled to capture this on MRI. This perivenous distribution was demonstrated *in vivo* using T2* imaging on a high field strength 7 T scanner,^27^ and this CVS was subsequently shown to effectively distinguish demyelinating lesions from asymptomatic white-matter lesions (such as those caused by microvascular disease).^28^ In a diagnostic analysis in a cohort of patients where there was uncertainty about the diagnosis, all the patients who went on to have a clinically confirmed diagnosis of multiple sclerosis had a visible central vein in the majority of their baseline brain lesions; by contrast, all those patients who did not receive a multiple sclerosis diagnosis only had a central vein visible in a minority of lesions.^29^ A subsequent analysis showed similar results when using a lower 3 T MRI field strength,^30^ and another demonstrated the sign’s ability to discriminate between other inflammatory CNS vasculopathies.^31^ Indeed, the ability of 1.5 T MRI to identify CVS on susceptibility-weighted imaging has been demonstrated in a number of studies,^32-34^ including one in the paediatric-onset population.^35^

A drawback of this approach is the need to count the total number of brain lesions and lesions with a central vein—a time-consuming endeavour when there are many lesions. Analyses have been performed on multiple simplified rating schemes: using a threshold of 35% of lesions showing a central vein or an absolute count of three or six lesions with the CVS.^36,37^ Using a count of three CVS lesions, sensitivity was higher, whereas requiring six lesions with a CVS preserved more specificity. In the 2024 diagnostic criteria, the presence of six lesions with a visible central vein (the ‘Select 6’ rule) can make a diagnosis of multiple sclerosis when accompanied by either DIS as demonstrated by lesions in at least two typical regions, or where there are lesions in only one region when accompanied by DIT or positive CSF analysis.^1^

#### Paramagnetic rim lesions

Demyelinated lesions can be classified into distinct subtypes, depending on the chronicity of the lesion and the presence of active inflammation.^38^ Chronically active lesions (also termed slowly expanding or smouldering lesions) feature an inactive centre with a rim of activated microglia.^39^ These microglia are laden with iron, and can therefore be detected by appropriate susceptibility-sensitive imaging^40^; early studies noted this on 7 T MRI alongside histopathological correlation with lesional iron distribution,^41^ with further analyses confirming the ability of 3 T^42^ and 1.5 T^43^ scanners to adequately detect these paramagnetic rim lesions (PRLs). Longitudinal MRI analyses have also demonstrated that PRLs expand over time, whereas lesions without rims do not, suggesting they might contribute to disease progression.^44^

The association of PRLs with clinical outcomes has also been studied. The presence of PRLs is associated with greater disease severity and greater blood–brain barrier disruption.^45^ They are also shown to increase diagnostic specificity for multiple sclerosis, especially when combined with the CVS, and can predict conversion from a demyelinating clinically isolated syndrome to multiple sclerosis.^46^ Additionally, they are very rarely found in multiple sclerosis mimics, with the exception of Susac’s syndrome (though in lower proportions compared with multiple sclerosis).^47^

The new criteria propose that PRLs can be used to increase the specificity of a multiple sclerosis diagnosis. In patients presenting with their first demyelinating event but who only have lesions in one typical CNS location, if they have DIT on MRI (interval change or a mixture of gadolinium-enhancing and non-enhancing lesions) or OCBs in CSF, the presence of a PRL is sufficient to make a diagnosis of multiple sclerosis. However, in contrast to CVS use, the presence of a PRL is not a sufficient substitute for DIT or positive CSF if DIS is met by lesions in two typical CNS regions.^1^

### Kappa free light chain index as an alternative to OCB

As described earlier in this article, CSF examination for OCB continues to have a role in the diagnosis of multiple sclerosis. However, techniques for their detection are rater-dependent and limited to specialized laboratories. Following publication of the 2001 version of the diagnostic criteria,^5^ an expert group published a consensus statement on the recommended standard of CSF analysis in multiple sclerosis,^48^ to ensure the validity of OCB use when diagnosing multiple sclerosis. When performed correctly, according to the consensus statement, OCB testing has a sensitivity of 95% and specificity of 86% in the context of multiple sclerosis. Even in this context, inter-rater variability has been reported: an Italian group identified a large inter-laboratory variability in OCB pattern reporting and differentiating OCB-positive and OCB-negative samples,^49^ with much of the variability being ascribed to the participation of general laboratories (rather than those with expertise in CSF analysis). A Canadian analysis further noted significant variability in CSF OCB processing, interpretation and reporting,^50^ which included differences in the cut-off for how many CSF-specific bands were required to confirm OCB positivity, and how patterns of systemic inflammation or monoclonal gammopathy were reported.

A more accessible biomarker is the kappa free light chain (kFLC) index, which can be performed using cost-effective techniques to provide a quantitative result.^51-53^ A positive kFLC index in CSF is associated with increased likelihood of further clinical events in early clinically isolated syndrome and multiple sclerosis, and predicts new T2-weighted MRI lesions in clinically and radiologically isolated syndromes.^54,55^ Concordance between OCB and a positive kFLC index is high: it has been reported as 94.2% in a population in whom multiple sclerosis is being considered,^56^ around 87% in a clinically isolated syndrome cohort^57^ and 90% in a primary progressive multiple sclerosis population.^58^ A systematic review and meta-analysis^52^ calculated a weighted average kFLC index sensitivity of 88% and specificity of 89%, with an OCB average sensitivity of 85% and specificity of 92%; there was no significant difference in diagnostic accuracy between the two measures. However, as there is no comparison with serum biomarkers, the kFLC index cannot differentiate between isolated intrathecal inflammation and systemic inflammation with additional intrathecal inflammation.^59^ The new diagnostic criteria propose that the kFLC index is an equivalent test to CSF-restricted OCB—both can now be used to demonstrate positive CSF analysis.^1^

It should be noted that, though both techniques are effective at identifying intrathecal immunoglobulin synthesis, the synthesis of such immunoglobulins is not a process specific to multiple sclerosis. Intrathecal immunoglobulin synthesis is seen in up to 43.5% of myelin oligodendrocyte glycoprotein (MOG) antibody-associated disease, up to 40% of neurosarcoidosis and in multiple autoimmune encephalitides (including up to 79% of anti-*N*-methyl-D-aspartate receptor encephalitis).^60^

### Unifying the multiple sclerosis spectrum under one set of criteria

Previous iterations of the criteria have not included the radiologically isolated syndrome, and have considered primary progressive multiple sclerosis separately. They have also excluded paediatric-onset multiple sclerosis from the criteria, and given no guidance (other than advising caution) in late-onset multiple sclerosis. These presentations have now been included in the latest criteria.

#### Broadening the phenotype

One of the themes of the new criteria is the move away from defining multiple sclerosis by clinical events, and more by its underlying biology. The previous focus on the clinical presentation meant that the radiologically isolated syndrome, in which MRI appearances are characteristic for demyelination in the absence of typical symptoms of multiple sclerosis,^61^ was not included in previous diagnostic criteria.

Radiologically isolated syndrome is often found incidentally on MRI studies performed for other indications. Various risk factors have been identified that predict likelihood of a future clinical event, including younger age, infratentorial or spinal cord lesions, lesions enhancing following gadolinium administration or positive CSF OCB—risk factors shared with those for the likelihood of developing a second clinical event after a clinically isolated syndrome, suggesting a similar underlying biology.^62,63^ Just over half of those diagnosed with radiologically isolated syndrome will go on to develop clinical symptoms within 10 years, with the risk increasing when more risk factors are identified.^64^ The CVS is also seen in radiologically isolated syndrome, with at least 40% of T2-weighted hyperintense lesions demonstrating the CVS.^65^ The new criteria propose that, in somebody with a radiologically isolated syndrome who has DIS, a diagnosis of multiple sclerosis can be made if they have either DIT, positive CSF or the presence of six CVS lesions.^1^

Additionally, as the clinical history of primary progressive multiple sclerosis was so distinct from relapse-onset multiple sclerosis, it had a separate subset of criteria alongside the main set for relapse-onset disease.^8^ There is now greater understanding that primary progressive multiple sclerosis is only quantitatively different to relapse-onset disease, rather than qualitatively distinct—underlying disease processes are similar, with the main differences seen in the timing and relative contribution of the degenerative rather than inflammatory components of the disease.^66^ Relapses are seen both in relapse-onset and primary progressive disease, and the onset of secondary progression in relapse-onset disease is similar to the onset of primary progression. Imaging features are also shared between the phenotypes, with accumulation of white-matter lesions on T2-weighted imaging, development of atrophy and enhancement of lesions following administration of gadolinium contrast (though this is less frequent in primary progressive disease compared with relapse-onset). Based on this understanding, an analysis assessed three modified versions of relapse-onset diagnostic criteria in a progressive-onset population: (i) the previous (2017) version of the diagnostic criteria for relapse-onset multiple sclerosis; (ii) a modification including the optic nerve in DIS; and (iii) by permitting two spinal cord lesions to fulfil DIS.^67^ Similar diagnostic performance was seen with all approaches compared with the previous gold standard, with sensitivity, specificity and accuracy all between 92% and 95%. The diagnostic flow chart for the new criteria therefore has as an entry point of either a typical demyelinating attack or objective progression over 1 year; the rest of the requirements for supporting paraclinical evidence are the same, other than the additional facility for DIS to be met with two or more spinal cord lesions in progressive-onset disease.^1^

#### All ages considered

Paediatric-onset and adult-onset multiple sclerosis share many clinical and pathological features,^68,69^ though paediatric-onset disease is characterized by more relapse activity and better recovery from an episode of demyelination.^70-72^ However, better recovery should not falsely reassure—progression independent of relapse activity is observed in paediatric populations,^71^ and early use of highly effective disease-modifying treatments appears to reduce the accumulation of disability.^70^ The previous iteration of the diagnostic criteria,^8^ while specifically not inclusive of paediatric-onset presentations, performed well in paediatric populations, with particular contribution noted from CSF examination.^73,74^

However, additional care needs to be taken in the paediatric population, as the differential diagnosis is distinct from that for adult-onset presentations. Specifically, the incidence of both acute disseminated encephalomyelitis (ADEM)^75,76^ and MOG antibody-associated disease^77,78^ is higher in children versus adults (there is overlap between the two conditions). Also, there are limited data on the applicability of some of the proposed changes to the paediatric population: CVS-positive lesions can differentiate multiple sclerosis from other paediatric conditions, but the ‘Select 6’ rule has not been assessed in younger patients^79^; similarly, while PRLs are seen in paediatric multiple sclerosis patients, their diagnostic performance has not been reviewed.^80^

Consequently, though the authors include paediatric-onset multiple sclerosis in the new diagnostic criteria, they do so with some specific caveats.^1^ First, they advise that CVS should only be considered when more than 50% of lesions show the sign; other than commenting that PRLs are understudied in paediatric presentations, the authors make no recommendations regarding their use (or non-use). In presentations of ADEM, a diagnosis of multiple sclerosis cannot be made unless there is clinical or radiographic evidence of disease activity more than 90 days after ADEM onset. Finally, they strongly advocate for MOG antibody testing in anybody presenting with CNS demyelination before the age of 12, and in people over the age of 12 with non-specific symptoms or symptoms suggestive of MOG antibody-associated disease.

Care also needs to be taken at the other end of the age spectrum. With advancing age, comorbidities accumulate and can mimic features of multiple sclerosis.^81^ Lesional distributions, considered typical for multiple sclerosis, have some overlap with other conditions (particularly small vessel disease, migraine and other inflammatory conditions)^82,83^—the exception is spinal cord lesions, which are not typically found in cerebrovascular disease. Positive CSF for OCBs or kFLCs can support a diagnosis of multiple sclerosis,^84^ and the CVS can differentiate demyelinating lesions from those caused by cerebrovascular disease.^85,86^ The authors of the 2024 criteria^1^ therefore recommend that, in individuals presenting either with relevant comorbidities (such as hypertension, dyslipidaemia, diabetes mellitus, smoking or vascular disease) or aged above 50, additional evidence for multiple sclerosis should be sought: either a spinal cord lesion, positive CSF analysis or the presence of the CVS to give greater diagnostic confidence.

## Issues raised by new criteria

### Optic nerve involvement

The inclusion of the optic nerve within DIS reflects diagnostic realism. Optic neuritis is a common and archetypal entry point into multiple sclerosis,^87^ and modern imaging and electrophysiology allow objective confirmation of optic nerve involvement.^20-23^ Yet the optic nerve is not pathognomonic territory. Inflammatory, infectious, antibody-mediated and compressive optic neuropathies can all produce structural and functional abnormalities.^88,89^ Because the optic nerve can now complete DIS in otherwise borderline cases,^1^ it becomes a high-leverage region. A single additional region carries disproportionate diagnostic weight. The central issue is therefore not whether the optic nerve should be counted, but under what conditions. Variability in orbital MRI protocols, OCT interpretation and VEP performance risks heterogeneity in what is accepted as ‘optic nerve involvement’. In atypical optic neuritis phenotypes, particularly severe, bilateral, longitudinally extensive or recurrent presentations, alternative inflammatory disorders—such as neuromyelitis optica spectrum disorder, MOG antibody-associated disease or neurosarcoidosis—remain more plausible than multiple sclerosis.^89^ Without explicit safeguards, a sensitivity-enhancing change could create a new pathway to overdiagnosis.^90^

### Dissemination in space alone

Permitting diagnosis in the absence of DIT when DIS is sufficiently extensive represents a philosophical change. Historically, DIT has functioned as a behavioural safeguard, forcing clinicians to observe evolution before attaching a durable diagnostic label. Removing DIT in selected circumstances assumes that lesion specificity—secured by pattern recognition and, where available, advanced markers such as the CVS—can substitute for temporal confirmation. The argument that extensive spatial involvement confers very high specificity is persuasive in well-phenotyped cohorts with high-quality imaging. However, real-world populations include patients with vascular disease, migraine, systemic inflammation and neurosarcoidosis, all of whom may accumulate multifocal abnormalities on MRI.^91^ Time has traditionally filtered out mimics whose clinical or radiological course diverges from multiple sclerosis and diminishing its role risks accelerating premature diagnosis if specificity tools are applied inconsistently. The concern is not that DIT lacks value, but that it has served as a behavioural brake. Removing it in certain contexts assumes that lesion specificity can be secured by pattern recognition and, where needed, advanced markers.^25^ That assumption will only hold if those markers are applied rigorously and if clinicians resist the temptation to treat lesion count alone as sufficient.^90^

### MRI markers

The incorporation of the CVS and PRLs moves the criteria closer to lesion biology. This is intellectually coherent and potentially transformative. Yet these markers are technically and interpretatively demanding. Their performance depends on field strength, sequence optimization, lesion selection rules and reader training. Thresholds, such as a minimum number of lesions with a visible central vein,^37^ are only as robust as the denominator and the acquisition quality.

For these biomarkers to improve diagnostic accuracy at scale, they must be translated from research features into repeatable clinical measurements. The operational layer is frequently underspecified. Questions that determine real-world performance are often left implicit: who interprets the sign, on which sequence, using what lesion inclusion rules, against what denominator and with what statement of uncertainty. Without explicit agreement on these elements, supportive biomarkers risk functioning as rhetorical reinforcement rather than as reproducible measurements that can be audited and standardized.

For CVS, at least three operational decisions materially affect performance. First, lesion selection. Counting every punctate T2 focus inflates uncertainty and may distort apparent proportions, whereas selecting only larger lesions risks bias towards classic periventricular plaques. Second, the denominator. Proportion-based thresholds require a minimum number of assessable lesions to avoid spuriously high percentages derived from small samples. Third, reader training and agreement. The central vein can be subtle,^92^ particularly on suboptimal sequences, and inter-rater variability is non-trivial without structured training and consensus rules. These are not peripheral technicalities; they determine whether CVS can function as a specificity tool outside expert centres.^30,37,93^

PRL is even more sensitive to acquisition and definition. Susceptibility contrast depends on sequence choice (including T2*-weighted and susceptibility-weighted imaging), spatial resolution and post-processing.^40^ The North American Imaging in Multiple Sclerosis Cooperative consensus provides radiological criteria and explicitly acknowledges unresolved issues, including how to handle mixed or partial rims, susceptibility artefacts, and the relationship between rim lesions and slowly expanding lesions.^94^ In routine reporting, reporting of a PRL may benefit from structured reporting of acquisition parameters and an explicit confidence statement. A useful analogy is gadolinium enhancement: radiologists do not declare enhancement without specifying contrast use and sequence context. Similarly, standardization of PRL reporting with susceptibility context may strengthen confidence in its use.

The risk, therefore, is not conceptual but operational. Under controlled research conditions, CVS and PRL demonstrate impressive discriminatory power. In routine practice, where scans may be motion-degraded, sequences incomplete and reporting unstructured, these markers could be applied inconsistently, undermining their intended role as specificity safeguards. Uneven access to susceptibility-sensitive imaging further increases the likelihood of a two-tier diagnostic landscape, in which specificity tools are available in principle but variably realized in practice.

### Kappa free light chains

Recognizing the kFLC index as equivalent to CSF-restricted OCBs improves accessibility and standardization. Measurement of kFLC can be automated, yields continuous quantitative values and may shorten turnaround times, making it attractive for scalable implementation. That said, operational and financial barriers to its implementation are not insignificant—centres already offering CSF OCB analysis may not wish to change pathways, and centres not already performing CSF analysis for multiple sclerosis will have to invest in new equipment and skills for staff.

Additionally, intrathecal immunoglobulin synthesis remains a probabilistic marker, not a disease-specific one. The use of kFLC intensifies a familiar cognitive hazard: a positive CSF marker can be used as a shortcut to certainty. In misdiagnosis cohorts, clinicians have treated intrathecal synthesis as proof that MRI lesions must therefore represent multiple sclerosis, when the correct logic runs in the opposite direction. Intrathecal synthesis raises probability only when pre-test probability is already reasonable—that is, when the clinical syndrome and MRI pattern are genuinely multiple sclerosis-consistent.^12,90^ In older patients with vascular risk or in those with systemic inflammatory disease, a positive kFLC may not carry the same specificity and, if used indiscriminately, could worsen overdiagnosis. OCBs may be more helpful in this context, where the differential diagnosis is broad and includes other autoimmune conditions, and kFLC may be less able to distinguish these from multiple sclerosis.^59,95^

Standardization is also a non-trivial issue. The kFLC index depends on paired serum measurement, assay calibration and correction for blood–CSF barrier integrity. Thresholds are not universal. If kFLC is to substitute for OCB in routine pathways, laboratories and clinicians will require agreed reporting conventions, reference ranges, and explicit statements about what a given result can and cannot imply.^59^

### Equity

Every revision implicitly sets an equipment standard. The 2024 McDonald diagnostic criteria^1^ increase the diagnostic role of MRI and introduce advanced imaging markers that require optimized iron-sensitive sequences. In well-resourced centres this is feasible; in many settings it is not.

The risk is a two-tier diagnostic landscape. One tier can deploy CVS and PRL to enhance lesion specificity and reduce misdiagnosis. The other must rely on conventional MRI and CSF markers and may therefore either underdiagnose out of caution or overdiagnose because specificity safeguards are unavailable. The MRI consensus recommendations^92^ attempt to mitigate this by distinguishing core diagnostic sequences from optional advanced acquisitions and by emphasizing quality assurance but guidance alone does not eliminate structural disparity.

It should be noted, however, that there is also inequity in continued reliance on lumbar puncture in centres without access to appropriate MRI facilities. Where diagnostically equivalent alternatives are available, patients generally prefer non-invasive investigations over lumbar puncture, reflecting the procedure’s associated discomfort, adverse effects and recovery time.^96^ The procedure is not without complications, even if they are mild or rare,^97^ and is associated with patient anxiety and diagnostic delay.

Implementation will therefore require explicit health-system choices. In regions without routine access to iron-sensitive imaging, should equivocal cases be referred to tertiary centres for diagnostic MRI, or will lumbar puncture continue to be used? Should spinal cord imaging be prioritized to strengthen DIS using conventional tools? Should kFLC be adopted to streamline CSF workflows where laboratory infrastructure allows? These are not abstract technical questions. They determine whether the 2024 revision narrows or widens diagnostic inequities—this will be particularly relevant in regions with lower rates of multiple sclerosis and higher prevalence of nutritional, inflammatory and infectious mimics.^98^

## Implications for real-world use

### Embedding optic nerve inclusion within disciplined pathways

In practice, optic nerve involvement should be credited only when two conditions are met: (i) the clinical syndrome (or CNS imaging in the case of radiologically isolated syndrome) is objectively typical for demyelinating disease; and (ii) objective evidence supports intrinsic optic nerve pathology. Where phenotypes are atypical, antibody-mediated disorders should be actively considered before criteria are applied and documentation of why alternatives are unlikely should be routine rather than implicit. Operationally, centres should define what constitutes an adequate orbital protocol if optic nerve involvement is to be used diagnostically. MRI, OCT and VEP may support clinical assessment but should not substitute for diagnostic reasoning. The optic nerve should strengthen a typical presentation, not rescue an atypical one.

### Behavioural safeguards when DIT is absent

Where diagnosis is made based on extensive DIS without DIT, explicit confidence grading should become routine. A recorded judgement of high, moderate or low confidence forces clinicians to state why the case is being treated as multiple sclerosis now and creates a clear trigger for reassessment when confidence is incomplete. This matters most in the ‘grey zone’, where symptoms are non-localizing and imaging is persuasive but not specific. In these settings the criteria should not be treated as a screening tool. A practical workflow is to classify the clinical syndrome before reviewing imaging, then assess MRI using a structured approach that foregrounds morphology, distribution and red flag features, and only then interpret CSF and advanced markers. DIS should not become a lesion count exercise. Pattern recognition and exclusion of mimics remain the core of specificity.

### Translating MRI biomarkers into reproducible practices

If the CVS and PRLs are to improve accuracy outside expert centres, they must be operationalized as measurements, not motifs. In routine services, the safest way to do this is to distinguish routine MRI from diagnostic MRI. CVS and PRL should be treated as escalation tools for equivocal but typical cases, deployed with defined acquisition parameters, standardized reporting and a statement of confidence. Opportunistic interpretation on whatever sequences happen to be available risks turning supportive biomarkers into inconsistent opinions.

Debates about 1.5 T versus 3 T can obscure what matters more (many regional centres still routinely use 1.5 T for diagnostic imaging). Completeness and consistency of protocol, quality assurance and standardized reporting often determine diagnostic value at least as much as field strength. A diagnostic MRI for suspected multiple sclerosis should reliably cover the brain and spinal cord; include sequences that are sensitive to periventricular and cortical or juxtacortical lesions such as 3D fluid-attenuated inversion recovery; include T2-weighted imaging; and document gadolinium use with a clear rationale.^92^ Susceptibility sequences become strategically important in the current era even if they are not acquired universally. A centre does not need to perform CVS or PRL assessment on every patient, but it should have an accessible pathway for obtaining susceptibility-enabled imaging when lesion specificity is uncertain. The biggest avoidable error is treating incomplete or low-quality imaging as definitive. If spinal cord imaging is missing in a myelopathic presentation, if cortical coverage is inadequate or if motion degrades interpretability, the correct response is to repeat imaging under diagnostic conditions rather than apply the criteria anyway. Specificity is frequently lost to protocol shortcuts, not to the criteria themselves.

### Using kappa free light chain index

The kFLC index should be integrated into practice as a supportive test within a structured diagnostic sequence, not as an independent trigger for diagnosis. In routine care this means deferring CSF interpretation until the clinical syndrome and MRI pattern have been judged compatible with demyelination, and explicitly recording how the kFLC result modifies diagnostic confidence. Laboratories can contribute by standardizing reporting, including paired serum values, reference ranges and brief interpretative comments regarding blood–CSF barrier function. Clinicians, in turn, should avoid binary use of the result. The same kFLC value carries different implications in a young patient with a typical syndrome and characteristic lesion morphology than in an older patient with vascular risk and non-specific symptoms. Embedding kFLC within a structured workflow reduces the risk that it becomes a shortcut to certainty rather than a refinement of probability.

### Diagnostic criteria, not management guidelines

One of the consequences of the new criteria is the possibility of diagnosing otherwise asymptomatic radiologically isolated syndrome as multiple sclerosis.^1^ This said, though the radiologically isolated syndrome is characterized by a lack of clinical relapses,^61^ there is evidence to suggest there is a multiple sclerosis ‘prodrome’, reflected by increasing healthcare use and higher rates of psychiatric symptoms^99^—the lack of classical relapses is not equivalent to lack of disease effect. This allows the criteria to acknowledge the underlying biology of multiple sclerosis, and not just its clinical manifestations. Indeed, this reflects and extends the role that previous iterations of the criteria played in incorporating clinically isolated syndrome into the multiple sclerosis spectrum when there was sufficient supportive paraclinical evidence.

The criteria themselves do not address whether individuals with radiologically isolated syndrome who now meet diagnostic thresholds for multiple sclerosis should receive disease-modifying therapy—they are a diagnostic rather than therapeutic document. Data in this area are limited, but not absent. Two randomized controlled trials have been carried out in radiologically isolated syndrome: one comparing dimethyl fumarate with placebo^100^ and the other teriflunomide with placebo.^101^ Both demonstrated a reduced rate of first clinical demyelinating event in the treatment arm over 2 years, as well as a good safety profile for both interventions. However, the trials were of low numbers with high drop-out rates. While longer-term trials have not been carried out, and there are no data for higher-efficacy treatments, the trials serve as proof of concept that disease-modifying treatments may have a role to play in management of radiologically isolated syndrome. As early treatment of multiple sclerosis is one of the key factors in limiting accumulation of disability,^9^ it will be important to investigate the role that higher-efficacy treatment can have on radiologically isolated syndrome diagnosed as multiple sclerosis (a trial of ocrelizumab in radiologically isolated syndrome was terminated early due to slow recruitment).^102^

This therefore raises an ethical boundary between defining disease and defining treatment eligibility. While randomized trials demonstrate that disease-modifying therapy can delay a first clinical event in radiologically isolated syndrome, a 2-year delay is not equivalent to proven long-term disability reduction, and the safety calculus differs fundamentally in asymptomatic individuals. With radiologically isolated syndrome able to be reclassified as multiple sclerosis based on biomarkers alone, the term will expand to a biological definition rather than its traditional clinical meaning. Without careful consideration of thresholds for treatment, diagnostic inflation at this boundary could expose individuals to long-term therapy, insurance consequences and psychological burden without definitive evidence of sustained benefit (or indeed delayed treatment and accumulation of avoidable disability). This is likely to vary internationally if local treatment protocols restrict prescriptions of disease-modifying treatment to those with evidence of clinical disease activity. Accordingly, biomarkers in radiologically isolated syndrome should inform both risk stratification and consideration of early intervention, while acknowledging ongoing uncertainty regarding long-term treatment benefit.

### Addressing inequity

The 2024 McDonald diagnostic criteria are likely to be implemented unevenly. Some centres will have ready access to susceptibility-enabled MRI and neuroradiology expertise; others will not. The practical question is therefore how to preserve diagnostic safety when only the core tools are available.

A workable approach is to make equity an explicit design goal of local pathways. First, define a minimum diagnostic imaging standard that can be delivered reliably, independent of field strength and do not apply the criteria when that standard is not met. Second, create an escalation route for equivocal but typical cases, such as referral for a diagnostic MRI that includes susceptibility sequences or specialist neuroradiology review, rather than relying on opportunistic interpretation of suboptimal scans. Third, prioritize spinal cord imaging where clinically indicated, because completing an adequate conventional work-up often improves specificity more than adding novel markers. Fourth, adopt kFLC where assays are locally validated and reporting is standardized, but embed it within the same structured workflow and confidence grading used for OCB, rather than treating it as a substitute for imaging quality.

Finally, monitor equity as an outcome. Services should audit rates of diagnostic revision, misdiagnosis, and time to definitive diagnosis by referral route and by access to advanced imaging. The ethical test of the 2024 McDonald diagnostic criteria is not whether it can accelerate diagnosis in the best-equipped centres, but whether it can be implemented safely without widening disparities.

### A unifying principle

The 2024 criteria bring diagnosis closer to the biology of multiple sclerosis and closer to (or even before) the first clinical event. Their real-world impact will therefore be determined less by what the criteria permit than by how they are used. Misdiagnosis is best understood as a systems problem created by incentives and uncertainty. Clinicians feel pressure to provide an explanation, patients seek closure and MRI delivers visually persuasive abnormalities even when these are non-specific. In this context, additional biomarkers can either improve diagnostic precision or supply new props for unwarranted certainty.

The practical safeguard is to treat the criteria as a structured decision pathway, not as a checklist. This requires two features: first, the criteria should be applied only after the clinical syndrome has been classified, because typical syndromes define the appropriate pre-test probability; second, the output should include an explicit confidence grade and a documented mimic check. Where certainty is incomplete, the correct response is not to force a binary diagnosis but to state uncertainty and commit to reassessment if evolution fails to match expectation.

Diagnostic language matters because it determines downstream consequences. A categorical multiple sclerosis diagnosis triggers durable labels and treatment cascades. Vague language such as possible multiple sclerosis is a retrograde step and can generate repeated imaging and follow-up without a plan. Structured clarity is more useful than either certainty or ambiguity. Documentation can separate three statements: whether the syndrome is typical for demyelination, whether MRI is multiple sclerosis-consistent and why, and whether CSF and MRI biomarkers support chronicity or specificity. Where confidence is not high, that should be explicit and paired with a defined reassessment plan. Language that preserves uncertainty prevents the commonest cascade error in this field, turning uncertainty into certainty by omission.

### A stepwise workflow

The highest-risk moment in routine neurology is often the first consultation after a brain MRI has already been performed. The scan is present, symptoms may be non-specific and the clinician feels pressure to decide whether the MRI explains everything.

An alternative interpretation of the 2024 criteria is that increasingly specific biomarkers may permit diagnosis in selected atypical presentations when the biological signature of multiple sclerosis is sufficiently convincing. While current evidence remains incomplete, future iterations may move towards parallel diagnostic pathways in which clinical presentation and biomarker specificity contribute jointly rather than hierarchically to diagnostic confidence. In the meantime, the criteria will not prevent misdiagnosis unless embedded within a workflow that makes anchoring difficult. A practical six-step sequence (visualized in Fig. 1) for a symptom-first presentation is:

- Classify the clinical syndrome before reviewing the MRI as typical or atypical for demyelination.
- If there are any red flags, consider an enhanced diagnostic pathway incorporating more stringent imaging and biomarker criteria before applying the diagnostic framework. Generate a mimic-first differential and pursue targeted testing instead.
- Assess MRI using a structured template that prioritizes lesion morphology, distribution and red flag features rather than raw lesion counts.
- Use CVS and PRL as escalation tools for equivocal but typical cases or atypical cases, ideally on diagnostic-quality susceptibility-enabled imaging or with specialist neuroradiology review, rather than opportunistic calls on routine scans.
- Interpret CSF only after imaging has been judged consistent with multiple sclerosis (including, were available, advanced imaging in step iv). OCB or kFLC should support a diagnosis in the right context, but should rarely be decisive when MRI is non-specific.
- Record diagnostic confidence and the mimic check explicitly, and commit to reassessment when confidence is not high, with a defined follow-up interval or trigger.

**Figure 1:**
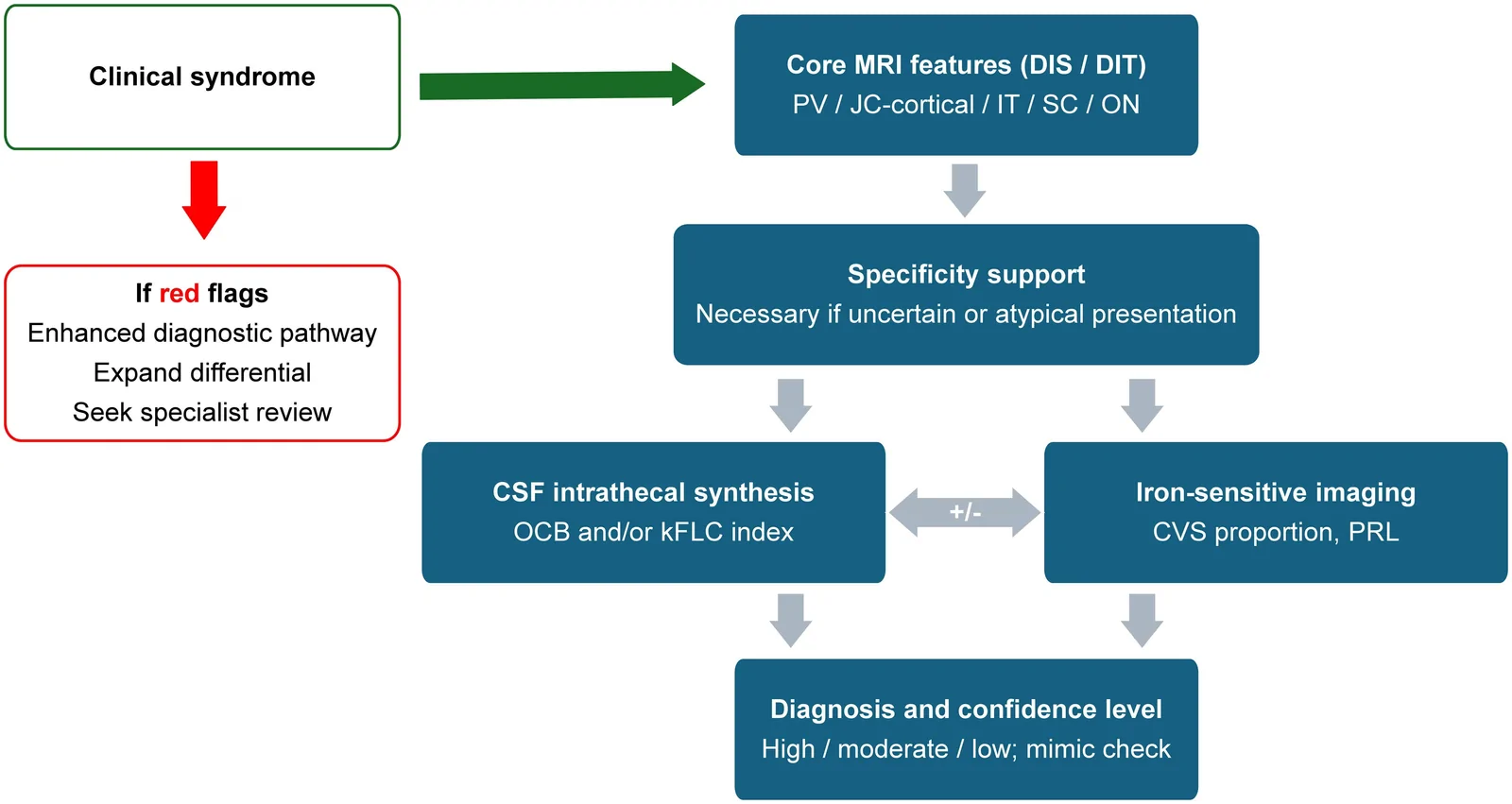
**Proposed diagnostic workflow under McDonald 2024.** CVS = central vein sign; DIS = dissemination in space; DIT = dissemination in time; IT = infratentorial; JC = juxtacortical; kFLC = kappa free light chain; OCB = oligoclonal bands; ON = optic nerve; PRL = paramagnetic rim lesion; PV = periventricular; SC = spinal cord.

This workflow makes explicit what the criteria assume but do not enforce: that pre-test probability must be set by the syndrome, that MRI interpretation must be structured, that biomarkers should modify confidence as tools to enhance specificity rather than determine diagnosis and that uncertainty should be documented rather than hidden.

## What’s next?

Every revision is followed by a period of living validation in which unanticipated benefits and harms become visible. If the criteria are used as intended, they will likely accelerate diagnosis in optic neuritis-predominant entry points and reduce misdiagnosis when CVS and PRL are used to enhance specificity. If used poorly without safeguards, they may instead accelerate misdiagnosis. The next iteration should prioritize operational science over conceptual novelty, including field-strength-robust CVS thresholds with training curricula, reproducible PRL definitions with clear evidence of incremental diagnostic value and implementation guidance that explicitly addresses settings without routine access to susceptibility imaging. It should also treat uncertainty as a criterion feature rather than a failure, by embedding confidence grading and explicit ‘do not apply’ statements for atypical syndromes.

## Conclusion

The 2024 McDonald diagnostic criteria are a serious attempt to modernize multiple sclerosis diagnosis by integrating biomarkers closer to disease biology and by responding—implicitly—to misdiagnosis harms. Its success will depend on whether clinicians treat it as a framework for disciplined earlier diagnosis in typical cases, rather than as a permissive licence to diagnose based on incidental imaging plus a positive CSF test. For *Brain*’s readership, the most provocative implication is this: specificity is no longer guaranteed by lesion location alone. It must be earned—by quality, by standards and by explicit attention to where we can fool ourselves.
